# Associations between internet addiction and academic stress among college students: a sequential mediation model involving social anxiety and self-control

**DOI:** 10.3389/fpsyg.2026.1737711

**Published:** 2026-09-21

**Authors:** Jin Jiang, Hao Wang, Zhihan Chen, Xiaoyao Zhang, Chengqi Ye

**Affiliations:** School of Environment and Energy Engineering, Anhui Jianzhu University, Hefei, China

**Keywords:** academic stress, college students, Internet addiction, self-control, social anxiety, sequential mediation model

## Abstract

**Objectives:**

To explore how Internet addiction is associated with college students’ academic stress and how self-control and social anxiety play a mediating role in this relationship.

**Methods:**

Using stratified sampling, 230 college students aged 18–22 years old from multiple universities in Hefei, Anhui Province, were recruited. All participants completed four standardized questionnaires. Serial mediation analysis based on SPSS PROCESS Model 6 with bias-corrected percentile Bootstrap (5,000 resamples) was adopted to test the hypothesized mediating effects.

**Results:**

The findings of this research indicate that Internet addiction is correlated with multiple indicators of college students’ academic stress. (1) Self-control and social anxiety each statistically mediate the association between Internet addiction and academic stress. (2) Social anxiety and self-control form a sequential pathway that statistically mediates the association linking Internet addiction and academic stress.

**Conclusion:**

These correlational results suggest that greater offline social engagement and stronger self-control may be related to lower levels of academic stress and less Internet addiction among university students.

## 1 Introduction

As Internet technology advances by leaps and bounds and smart devices become popular on a large scale, the Internet is the primary source for college students to obtain information, socialize with others, and entertain themselves. According to the 53rd Statistical Report on Internet Development in China, by the end of 2023, the number of Internet users in China had climbed to 1.092 billion [China Internet Network Information Center (CNNIC), 2024]. According to the 2024 report released by the China Internet Network Information Center, the data indicates a significant increase in Internet penetration rates. Over the preceding ten-year period, the total Internet user count in China has grown at a rate of more than 5 percent every year. Even though the Internet offers numerous benefits, the psychological and physical adverse outcomes associated with excessive or maladaptive Internet use have garnered widespread attention from psychologists and educators. As a phenomenon of psychological dependence, it has become one of the most common disturbances in the college student populations. According to statistics, around 11% of Chinese college students suffer adverse consequences from Internet addiction (Qiu et al., 2023). Lu et al. (2023) summarized the characteristics of Internet addicts across physiological, psychological, and social dimensions, a framework that lays a theoretical foundation for the current study’s hypotheses regarding academic stress, social anxiety, and self-control. Physiologically, prolonged online immersion triggers short-term intense excitement and disrupts bodily biochemical functions such as dopamine balance; such persistent physiological overstimulation may be related to depleted self-control resources, which could correlate with poorer restraint of impulsive online behavior and reduced academic energy, and may be linked to higher self-reported academic stress. Psychologically, addicts gain pleasure from repeated online engagement yet endure withdrawal distress offline, driving them to rely on virtual interactions to escape uncomfortable real-world social scenarios. This chronic offline avoidance is associated with interpersonal cognitive bias and higher social anxiety symptoms. Socially, excessive Internet use breeds apathy toward face-to-face communication, deepening social isolation. Elevated social anxiety may correlate with further reductions in learning-related self-control resources, forming a sequential correlational pathway: Internet addiction→social anxiety→lower self-control→higher academic stress. Collectively, the physiological, psychological, and social correlates of Internet addiction are jointly associated with higher academic stress, and highlight the necessity of targeted interventions to safeguard college students’ physical and mental well-being.

Academic stress is defined as stress originating from learning activities and the school adaptation process, such as high expectations from parents, strict demands from teachers, competitive pressure among peers, and self-imposed high standards, which collectively constitute a heavy psychological burden and tension for students (Chandra, 2021). In a context where academic achievement is closely related to social status, students’ academic performance is highly scrutinized, which undoubtedly further aggravates their academic stress. High self-reported academic stress is associated with greater anxiety, depressive symptoms, and reality-avoidant behaviors. Escape from self theory points out that the primary source of Internet addiction is imbalance between the individual’s ideal self and the real self (Cheng and Lin, 2023). When individuals face overwhelming self and environmental expectations, ideal-real self-discrepancy is associated with lower self-worth alongside negative emotions such as depression, anxiety, and guilt, accompanied by depression, anxiety, guilt and other negative emotions. In this case, individuals tend to escape the imbalance between ideal and reality and strong negative emotions by indulging in online games. academic stress, as a key environmental correlate, is associated with greater dissonance between ideal and real selves. When students face great academic stress, it is difficult for them to achieve the ideal academic goals set by themselves or others, so they feel the gap between their ideal and real selves, and then they develop negative emotions and eventually escape from reality through Internet addiction. This avoidance behavior establishes a bidirectional vicious cycle rather than yielding a unidirectional outcome. Excessive internet engagement is correlated with poorer academic functioning via multiple statistical pathways: it consumes substantial amounts of study time, disrupts regular sleep patterns, undermines sustained concentration during both class and self-study sessions, increases procrastination, and diminishes learning efficiency. These cumulative academic obstacles correlate with larger gaps between actual and ideal academic outcomes, as well as greater perceived learning burden, self-doubt, and academic stress. In essence, Internet addiction is correlated with higher concurrent academic stress. Zhang et al carried out a longitudinal tracking investigation with four waves of data collection and cross-lagged analysis to investigate the correlation between academic stress and Internet addiction among college student populations. The findings demonstrated a statistically significant positive correlation between the intensity of academic stress and the degree of Internet addiction among participants. Furthermore, the longitudinal data demonstrated a bidirectional correlational pattern: academic stress was associated with later Internet addiction, and Internet use patterns correlated with subsequent academic stress. The findings of this study provide correlational evidence for a hypothesized cyclical association between academic stress and Internet addiction, further supporting the view that Internet addiction is statistically linked to high academic stress (Zhang and Chen, 2022). Therefore, this investigation proposes Hypothesis H1: Internet addiction is positively associated with academic stress among college students.

Social anxiety is a prevalent psychological condition that manifests itself as persistent worry or fear on the part of the individual in social situations, which in turn interferes with normal life and interpersonal engagements (Chen et al., 2020; Liu et al., 2024). Lai et al. (2015) found that social anxiety correlated with lower subjective well-being and may have been associated with problematic phone use, Internet addiction, and aggressive tendencies (Lai et al., 2015). Individuals who are addicted to the Internet typically exhibit more avoidant behaviors and negative emotions in interpersonal contexts. The accumulation of these negative experiences is associated with higher levels of offline social anxiety. Within the theoretical construct of the cognitive model pertaining to social anxiety, individuals with this condition tend to exhibit a cognitive bias characterized by the over-amplification of negative information, resulting in heightened sensitivity to interpersonal interactions (Hou et al., 2021), thereby diminishing opportunities for interpersonal interaction in real-world settings, which correlates with more severe social anxiety symptoms. In such circumstances, those experiencing social anxiety often tend to rely on media such as mobile phone games and short videos to alleviate their inner social pressure and tension. Higher social anxiety symptoms are associated with poorer engagement with academic tasks, impaired concentration, and weaker emotion-behavior regulation; this correlational pattern links greater anxiety to higher perceived academic burden and more stressful learning experiences (Blackhart et al., 2015). In consequence, this study puts forward the hypothesis H2: Social anxiety statistically mediates the association between Internet addiction and academic stress among university students.

Self-control denotes an individual’s cognitive and behavioral capacity to deliberately manage impulsive actions and resist the urge to fulfill immediate wants and needs, such as resisting temptation, delayed gratification and impulse inhibition (Zhang and Gao, 2023; Li and Jin, 2020). Based on the self-control reward model, individuals must continuously strive to control their behavior in order to achieve long-term goals. However, when reward gain and effort expenditure are mismatched, poorer self-control may be observed (Rafieian and Sharif, 2023). Compared with ordinary individuals, Internet addicts need to make efforts to suppress negative emotions, but the results are often not satisfactory, which is further associated with their poorer executive function. Research shows that the strength of self-control capacity is significantly associated with the degree of individual dependence on the network. The self-regulation theory points out that maladaptive behaviors are strongly correlated with self-regulation deficits (Peng et al., 2020). Self-regulation deficits are associated with difficulty restraining online cravings and higher levels of Internet addiction. Excessive internet usage is associated with greater depletion of the finite physiological and psychological self-regulatory resources. Prolonged excessive Internet use is correlated with depleted self-regulatory resources, and lower self-control is associated with poorer academic time management, greater procrastination, and higher academic stress. Li et al. (2019) revealed that college students with higher self-control can better manage their online behaviors and avoid over-indulgence in the Internet, thus reducing academic stress (Li et al., 2019). Conversely, individuals with weaker self-regulation show greater Internet addiction, which correlates with poorer academic performance and higher academic stress. Additionally, Huang et al. (2022) found that stress is associated with Internet addiction both directly and indirectly via its statistical link with self-control as a mediator. This underscores the pivotal role of self-regulation in the association between stress and addictive behavior (Huang et al., 2022). In view of these observations this study suggests hypothesis H3: In the context of collegiate populations, self-control has been identified to exert a mediating influence in the association between Internet addiction and academic stress.

In addition, weakened self-control reduces an individual’s perceived self-efficacy and produces more negative emotions (Shen et al., 2023), thus a significant inverse relationship exists between self-control and social anxiety. The higher an individual’s level of social anxiety, the weaker their self-control abilities tend to be (Yang, 2022). Higher Internet addiction is associated with elevated social anxiety, which correlates with weaker self-control; poor self-regulation is linked to ineffective academic resource management, greater online avoidance of schoolwork, and higher perceived academic stress. Conservation of Resources Theory constitutes a psychological framework that explains the mechanism of stress perception formation (Hobfoll et al., 2016). The theory suggests that all prerequisites that can help people achieve their goals can be considered as resources. Individuals feel stress when they perceive that resources have been depleted, face potential depletion, or fail to obtain the expected return after resource depletion. For college students, social anxiety makes individuals feel uneasy and worried in social situations, depleting psychological resources; while impaired self-regulation capacity significantly compromises an individual’s ability to effectively inhibit prepotent responses and regulate impulsive behaviors in the face of salient temptations, depleting self-regulation resources. When college students exhibit excessive Internet usage, it can be associated with significant exhaustion of psychological and self-regulation resources, and this depletion does not bring academic rewards, but further correlates with higher levels of academic stress. Thus, it is evident that a strong correlation exists between Internet addiction, academic stress, social anxiety and self-control, which together affect individuals, but the specific correlational pathway linking these variables has not been clarified. Drawing on the Conservation of Resources Theory, this study proposes Hypothesis H4: The relationship between Internet addiction and academic stress is jointly mediated in a sequential manner by social anxiety and self-control. The study model is illustrated in Figure 1. It should be emphasized that the present study adopts a cross-sectional design. All hypothesized pathways described above represent correlational assumptions rather than definitive causal sequences.

**FIGURE 1:**
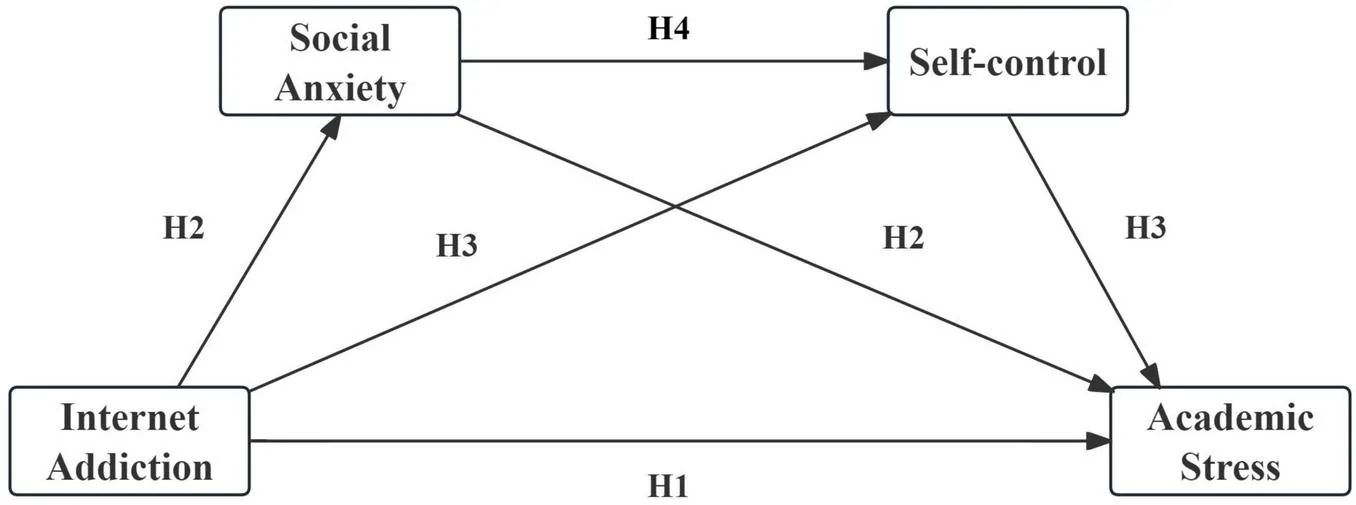
Research model.

Prior relevant studies mostly examined social anxiety and self-control as separate mediating variables in isolation. Distinctively, this research integrates the two variables within a unified sequential mediation framework to explore their serial transmission pathway. In summary, escape self-theory, self-regulation theory, and conservation of resources theory systematically elaborate the logical linkages among academic stress, social anxiety, self-control, and Internet addiction. These theoretical perspectives provide the core interpretive framework for the subsequent empirical findings of this study.

## 2 Materials and methods

### 2.1 Procedures and participants

Data for this study was collected through an online questionnaire. Before the commencement of the survey, all participants received official notification regarding the study’s rigorous privacy measures, ensuring personal data remained confidential. Each participant granted informed consent through the completion and signing of a consent form during class sessions. Subsequently, the questionnaire was disseminated to participants via the Questionnaire Star platform^1^, a tool utilized for the collection and secure storage of responses. Upon completion of data collection, all responses were systematically imported into SPSS for subsequent statistical analysis.

The participants of this study were recruited using stratified sampling, where universities served as the stratification variable. Five universities (Anhui Jianzhu University, Hefei Normal University, Hefei University, Anhui University, and Hefei University of Technology) were defined as independent strata. Given the comparable undergraduate population sizes across the five universities, 50 questionnaires were pre-allocated for each stratum. Within each university stratum, simple random sampling was implemented: we obtained undergraduate student rosters from each university, assigned unique random numbers to every eligible undergraduate, and selected study participants by random number drawing to avoid selection bias. In total, 250 questionnaires were distributed and collected across the five universities. After screening and removing invalid questionnaires (i.e., questionnaires with substantial missing values, logically contradictory responses, obvious careless filling, or completion durations less than 90 s), 230 valid questionnaires remained. The final valid sample sizes for each university stratum were 47, 46, 45, 46, and 46 respectively. The overall effective response rate was 92%. The participants ranged in age from 18 to 22 years (*M* = 20.46, SD = 1.72). For gender composition, 166 participants were male (72.4%) and 64 were female (27.6%).

### 2.2 Measures

#### 2.2.1 Internet addiction scale

In the survey, the degree of Internet addiction tendency among participants was assessed through a concise adaptation of the Internet Addiction Diagnostic Questionnaire originally formulated by Young (1996), which includes a total of 20 items (e.g., “Do you perceive that the duration of your online activities exceeds your initial expectations?”) (Cao, 2023), and measures from three dimensions: college students’ Internet time management, social interaction and interpersonal relationships, and emotional and psychological impacts. The measurement instrument employed in this investigation utilized a 5-point Likert scale, with response options ranging from 1 (denoting absolute non-compliance) to 5 (signifying complete compliance), and the validity of this scoring system has been fully substantiated by previous studies. In the present study, The mean values of participants’ ratings across all questionnaire items on this scale were utilized for analysis. The assessment results demonstrate that higher scale scores are positively correlated with increased susceptibility to Internet addiction. In addition, the overall α coefficient for the questionnaire reached 0.932, with the coefficients for each dimension being 0.881, 0.710, and 0.837, respectively, indicating high reliability of the scale.

#### 2.2.2 Academic stress scale

This study used the Academic Stress Perception Questionnaire (ASPQ) adapted by Chi Xiaojun for use with university students (Chi, 2017). The questionnaire consists of a total of 14 items, measuring from three aspects: examination and academic stress, learning efficiency pressure, and learning attitude and self-efficacy. The scale employed a five-point Likert-type scale for assessment purposes, where positive statements are scored from 1 to 5, ranging from “very non-compliant” to “very compliant,” and the reverse for negative statements. One of the questions, No. 14, was a reverse scoring question. A higher score indicates a greater sense of academic stress among the subjects. The internal consistency reliability of the questionnaire, as measured by Cronbach’s alpha, yielded a coefficient of 0.920 in the present investigation, and the split-half reliability was 0.877. The reliability coefficients for each dimension were 0.816, 0.821, and 0.817, respectively.

#### 2.2.3 Social anxiety scale

The present study employed Leary (1983) Social Anxiety Questionnaire as the measurement instrument to assess social anxiety levels among college student participants (Leary, 1983), which has 15 items in total assessing from two dimensions: anxiety in social situations and anxiety in communication and interaction. Elevated scores on this scale are indicative of greater severity of social anxiety symptoms in individuals. The scale has demonstrated strong reliability and validity among adolescent populations (You and Qin, 2020). The present investigation revealed a Cronbach’s alpha of 0.847 for the instrument, thus demonstrating strong reliability. The reliability coefficients for each dimension were 0.831 and 0.769 respectively.

#### 2.2.4 Self-control scale

The Self-Control Scale (SCS, 2021), which was adapted from the one revised by Tan Shuhua and Guo Yongyu, was used to measure the participant’s self-control at that time. The questionnaire consisted of 19 items, assessing from three dimensions: resistance to temptation and impulse control, behavioral habits and daily self-discipline, and emotional management and social impulse control. The self-control ability of participants was assessed through the computation of the mean score for each item within the scale. A higher score reflects stronger self-control and greater availability of self-control resources at that moment. In this study, the scale exhibited strong internal consistency, α = 0.894. The reliability coefficients for each dimension were 0.843, 0.856, and 0.812 respectively.

#### 2.2.5 Rationale for using composite total scores rather than separate dimension scores

All core hypotheses of the present study are constructed based on holistic associations between global internet addiction, academic stress, social anxiety and self-control, rather than independent correlational associations of each Internet addiction subdimension. The physiological-psychological-social three-dimensional framework mentioned in the Introduction is only adopted to interpret the overall formation mechanism of internet addiction, and our core research questions do not intend to compare the differences across its three sub-dimensions. In this context, analyzing composite total scores is more aligned with the core logical orientation of our serial mediation model. Although each scale has multiple sub-dimensions, the excellent overall Cronbach’s α coefficients of all four scales support the reliability of aggregating item scores into a unified total score to represent each latent psychological construct.

### 2.3 Statistical analysis

The collected data in this study were subjected to statistical analysis utilizing SPSS 26.0 software, with the chained mediation effect assessed through the SPSS macro program PROCESS. Prior to formal statistical analyses, univariate normality for all research variables was assessed via skewness and kurtosis statistics. The skewness coefficients across all variables ranged from -0.71 to 0.64, while kurtosis coefficients spanned -0.93 to 0.48. According to Kline (2023) criterion, absolute skewness values below 3 and absolute kurtosis values below 10 represent acceptable approximate normality; all coefficients in the present study satisfied this threshold, thereby validating the normality prerequisite for subsequent correlation and regression analyses. Pearson correlation analysis was adopted for bivariate correlation testing. This study delves into the mediating functions of social anxiety and self-control within the relationship between Internet addiction and academic stress. Finally, the serial mediating roles of social anxiety and self-control within that connection are examined using the SPSS PROCESS macro Model 6, with bias-corrected percentile bootstrap method adopted and 5,000 random resamples generated to test the significance of indirect effects.

## 3 Research findings and analyses

### 3.1 Common method bias test

The Harman single-factor test was employed to examine common method bias, and the results indicated that the Bartlett’s test of sphericity yielded a significant chi-square statistic. A total of 15 factors with eigenvalues exceeding 1 were identified, with the primary factor explaining 29.825% of the total variance, which falls below the 40% threshold commonly used as a critical benchmark. The absence of significant common method bias suggests that the measurement instrument employed in this investigation demonstrated adequate methodological rigor.

### 3.2 Descriptive statistics and correlation

Statistically significant positive correlations were identified through the correlation analysis between Internet addiction and both academic stress (*r* = 0.517, *P* < 0.01) and social anxiety (*r* = 0.467, *P* < 0.01). Conversely, a significant inverse relationship was observed between Internet addiction and self-control (*r* = −0.632, *P* < 0.01). Additionally, there exists a significant inverse relationship between academic stress and self-control ability (*r* = −0.556, *P* < 0.01). The findings derived from the initial statistical analyses are summarized and displayed in Table 1, with significant correlations between the variables at both the intra-individual and inter-individual levels. Thus, a basis for more in-depth examination of the chain mediation model was available.

**TABLE 1:** Descriptive statistics and correlation matrix of variables.

	*M*	*SD*	Skewness	Kurtosis	1	2	3	4
1 Internet addiction	2.034	0.678	0.58	−0.76	1	1	1	1
2 Academic stress	2.175	0.780	0.64	−0.52	0.517**			
3 Social anxiety	2.778	0.619	0.21	−0.93	0.467**	0.636**		
4 Self-control	3.145	0.683	−0.71	0.48	−0.632**	−0.556**	−0.573**	

*N* = 230, **P < 0.01, ***P < 0.001

### 3.3 A chain mediation test of social anxiety and self-control between internet addiction and academic stress

The correlation analysis results indicated that there was a certain association between social anxiety and self-control, which established a foundation for subsequent investigation into the mediating effect. Therefore, the research employed the bias-corrected percentile Bootstrap method and randomly selected 5,000 samples for analysis. During the analysis process, the SPSS plug-in Process developed by Hayes was utilized. This study considered Internet addiction as the predictor variable and academic stress as the outcome variable, and examined the potential mediating roles of social anxiety and self-control in this relationship. To explore the chain mediating effect among these variables, Process Model 6 was used for chain mediating test in this study.

Without considering the mediating variables, Internet addiction was positively correlated with academic stress (*r* = 0.517, *P* < 0.01). After introducing social anxiety and self-control as mediating variables, as shown in Table 2, Internet addiction was positively associated with social anxiety (β = 0.467, *P* < 0.01), while it was negatively associated with self-control (β = −0.466, *P* < 0.001). Even after the addition of the mediating variable, The positive association between Internet addiction and academic stress remained significant (β = 0.199, *P* = 0.011). Furthermore, social anxiety was negatively associated with self-control (β = −0.355, *P* < 0.001), exhibiting a considerable positive correlation with academic stress (β = 0.442, *P* < 0.001). On the contrary, self-control showed a significant negative correlation with academic stress. (β = −0.177, *P* = 0.036).

**TABLE 2:** Regression analysis of the chain mediation model between Internet addiction and academic stress.

	Social anxiety					Self-control					Academic stress				
	B	SE	β	t	*p*	B	SE	β	t	*p*	B	SE	β	t	*p*
Internet addiction	0.412	0.052	0.467	7.975**	< 0.01	−0.431	0.054	−0.466	−7.981***	< 0.001	0.185	0.072	0.199	2.569	0.011
social anxiety						−0.326	0.059	−0.355	−5.525***	< 0.001	0.403	0.075	0.442	5.373***	< 0.001
self-control											−0.162	0.077	−0.177	−2.104	0.036
R	0.467					0.706					0.694				
R^2^	0.218					0.498					0.482				
F	63.593					112.668					70.151				

*N*, 230; B, unstandardized regression coefficient; SE, standard error of unstandardized coefficient B; β, standardized regression coefficient.

By using the Bootstrap method, we conducted mediation effect tests and confidence interval analyses for the three mediating paths, and the findings are comprehensively presented in Table 3. The analysis of the mediating effects, as presented in Table 3, indicates that the 95% confidence intervals for all three mediating pathways exclude 0, which indicates that the indirect effects of all paths reached the level of statistical significance. Social anxiety and self-control are the mediators of the relationship between Internet addiction and academic stress, with a total standardized mediation effect value of 0.317, contributing to 61.32% of the total effect. This mediating effect was specifically composed of three different pathways, and their indirect effects each contributed 39.85%, 15.86%, and 5.61% to the total effect respectively. The specific model structure is shown in Figure 2.

**TABLE 3:** Mediated effects and confidence intervals.

Intermediary path	efficiency value	Proportion of total effect	95 per cent confidence interval	
			lower limit	limit
Internet Addiction→ Social Anxiety→ Academic Stress	0.206	39.85 percent	0.152	0.322
Internet Addiction→ Self-Control→ Academic Stress	0.082	15.86 percent	0.021	0.169
Internet Addiction→ Social Anxiety→ Self-Control→ Academic Stress	0.029	5.61 percent	0.006	0.062
Total indirect effect	0.317	61.32 percent	0.232	0.500

Indirect effects were estimated using 5,000 bias-corrected percentile bootstrap resamples.

**FIGURE 2:**
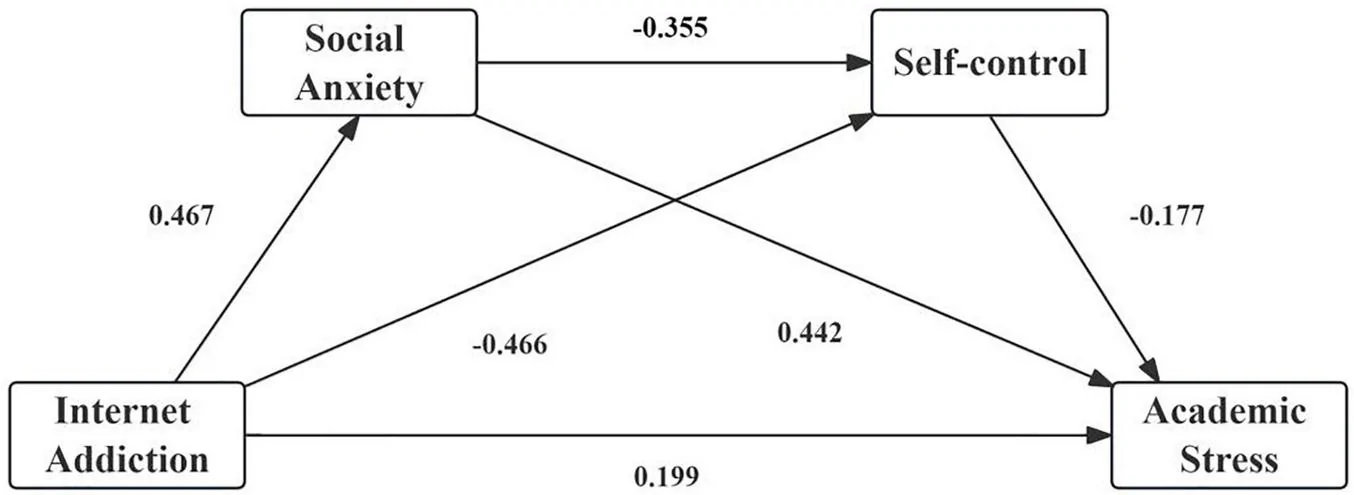
Diagram of the mediation effect model.

## 4 Discussion

This study examined the association between Internet addiction and academic stress among college students and tested whether social anxiety and self-control statistically mediated this association. A chain mediation model of “Internet addiction, social anxiety, self-control and academic stress” was constructed. Through regression analyses, a significant positive correlation was observed between Internet addiction and academic stress among college students. The findings from the mediation effect test indicated that social anxiety and self-control each served as partial mediators, and the chain mediation between the two, were significant.

### 4.1 Correlates linking internet addiction and academic stress

The study identified a notable positive association between academic stress and Internet addiction, which aligns with previous research (D’Souza et al., 2018). Higher self-reported academic stress correlates with multiple risk factors. Zhang et al. (2024) highlighted that a stronger tendency toward Internet addiction among college students may be associated with fewer resources allocated to study, which may in turn be linked to declines in academic performance and higher academic stress (Zhang et al., 2024). The behavior of over-reliance on the Internet tends to spend a lot of time in the online space, which not only takes away the time that individuals spend on studying, but also disrupts their normal work and rest routines (Qu et al., 2017). Such disruption of work and rest may be associated with reduced sleep quality and depleted energy for studying, and may therefore coincide with greater academic stress. In addition, higher Internet addiction is linked to poorer in-class and self-study concentration, which may in turn be associated with weaker individuals’ learning efficiency. Individuals exhibiting Internet addiction may also be more susceptible to academic procrastination and burnout than those in other groups (Zhang et al., 2022). These academic difficulties may be accompanied by greater perceived academic stress.

Notably, although the present research model specifies Internet addiction as the independent variable correlated with higher academic stress, this association is not strictly unidirectional. Academic stress may also be associated with increased Internet addiction. Severe academic stress may conversely also be associated with excessive online activities as an avoidant coping strategy. When students face overwhelming academic stress, unmet learning expectations, and persistent self-doubt, they may escape real-world academic frustration through anonymous online communication, gaming, and short-video browsing. Thus, this study supports an association between the two variables but cannot determine their temporal sequence or causal direction.

### 4.2 The mediating role of social anxiety

College students with elevated social anxiety suffer from persistent psychological distress in daily life. To evade the discomfort and evaluative threats inherent to offline interpersonal scenarios, they often isolate themselves from in-person gatherings and turn to online anonymous communication channels as a coping outlet (Lyvers et al., 2022; Dong et al., 2024). Such preference for virtual interaction is correlated with excessive online engagement and Internet addiction (Elhai et al., 2021). Greater Internet addiction correlates with gradual withdrawal from offline social interaction. This persistent disengagement is linked to fewer in-person opportunities to regulate anxiety, build academic resources, and gain social support, forming a correlational vicious cycle. Specifically, prolonged online avoidance correlates with worse social anxiety, which in turn may be associated with intensified Internet addiction and ultimately may contribute to elevated academic stress. Prior epidemiological evidence underscores the pervasiveness of social anxiety among undergraduates: more than half of college students report moderate social anxiety symptoms, and roughly one-third experience severe social-anxiety-related impairment, highlighting its critical role in shaping university students’ mental health (Yan et al., 2022). Consistent with the present findings, Huang et al. (2021) also documented a robust positive correlation between social anxiety and digital-device over-dependence. Excessive Internet use is correlated with disrupted offline social functioning and greater social anxiety; higher social anxiety links to poorer concentration, learning efficiency, and emotional regulation, all of which correlate with heavier perceived academic burden. In short, higher severity of Internet addiction is associated with aggravated social anxiety and elevated academic stress among college students. This serial correlational pathway extends Conservation of Resources Theory by illustrating continuous resource depletion correlated with online avoidance, and supplements the self-regulation perspective by revealing that poor offline adjustment is linked to maladaptive online coping and long-term negative academic correlates.

### 4.3 The mediating role of self-control

Baumeister relies on the finite resource model of self-control, (Baumeister et al., 2007) which proposes that self-control has the following properties: first, self-control is comparable to muscle power, an exhaustible and finite resource. This resource diminishes with sustained self-regulation effort, and resource depletion correlates with poorer self-control performance. Second, all self-control originates from the same source and follows the principle of conservation of energy, i.e., Ego depletion in one area may affect the depletion of other areas. Consequently, for college students, higher levels of Internet addiction are associated with depleted self-regulatory resources and weaker self-control. This makes them more prone to fatigue in the learning process, which in turn is linked to higher academic stress. As pointed out in the study by Song and Park, self-control has a significant impact on coping with stress and preventing Internet addiction. Hence, the correlational results of this study suggest that stronger self-control is statistically linked to lower academic stress and less Internet addiction (Song and Park, 2019). Consistent with their view, the present findings highlight that greater self-control correlates with lower academic stress and reduced Internet addiction. This finding aligns with the limited-resource model of self-control by showing that Internet addiction is correlated with diminished self-regulatory resources needed for consistent academic functioning.

### 4.4 Chain mediation of social anxiety and self-control

In addition to the exterior environment (the Internet), personal affective negativity (social anxiety) is correlated with reduced available self-regulatory resources (Blackhart et al., 2015). As a negative emotional condition, social anxiety is associated with fear and tension during social encounters (Zhang and Peng, 2023). This affective state correlates with greater consumption of self-regulatory resources to manage interpersonal discomfort. Research by Kashdan et al. (2011) indicates that individuals with higher social anxiety symptoms are associated with greater self-regulatory effort during social interactions compared to those with milder social anxiety. Blackhart et al. further elaborate that socially anxious individuals report faster consumption of self-regulatory resources during social interaction, which correlates with poorer self-regulation capacity (Blackhart et al., 2015). At the same time, learning behaviors require the consumption of large amounts of self-control resources. Thus, when self-regulatory resources are correlated with heavy online use and negative social affect, less capacity for academic self-regulation is linked to higher academic stress.

Therefore, interventions targeting improved self-control may correlate with lower academic stress and less Internet addiction. Physical activity, self-motivation, structured study schedules, and healthy daily routines may correlate with stronger self-control. In addition, social anxiety is negatively associated with social interactions, such as participation in club activities, group discussions and team projects, which are associated with mutual understanding and support among students. These measures provide a platform for them to release pressure and share experiences, which may be associated with lower levels of Internet addiction and reduced academic stress among college students.

### 4.5 Research significance

This study provides correlational theoretical and practical insights into factors linked to elevated academic stress among college students by testing the sequential statistical mediation pathways connecting Internet addiction, social anxiety, and self-control. Consistent with the proposed chain mediation model, Internet addiction was associated with higher academic stress both directly and indirectly through two parallel mediating paths (social anxiety, self-control) as well as one serial mediating path (social anxiety→self-control). Accordingly, interventions targeting social anxiety and self-control may correlate with lower Internet addiction and weaker statistical associations between Internet addiction and academic stress.

The correlational and mediating findings highlight the interconnected nature of Internet addiction, social anxiety, and low self-control, which may be associated with higher academic stress. Therefore, practical intervention strategies may prioritize reducing academic stress by targeting these two mediators, given their statistical links to Internet addiction. Two complementary intervention directions are proposed below. On the one hand, universities should implement systematic mental health interventions to reduce students’ social anxiety, which represents the initial link in the serial mediation chain. Schools can deliver regular mental health lectures and structured social skills training; such programs may correlate with greater interpersonal confidence and less offline social avoidance. Group counseling sessions provide a low-anxiety space for communication practice, which may correlate with less social avoidance and broader offline social support networks. In daily teaching, instructors should monitor students’ psychological states closely, identify early signs of severe social anxiety and deliver timely psychological support, which may correlate with weaker statistical links between social anxiety and academic stress. On the other hand, educational institutions and families should collaborate to strengthen college students’ self-control, a key downstream mediator linking social anxiety and academic stress. Universities can design self-regulation training modules covering time management, emotional regulation, and stress coping strategies. These self-regulation skills may correlate with less impulsive online behavior, greater cognitive investment in academics, and lower self-reported academic stress. Meanwhile, families also play an important role in supporting self-control; through daily supervision and positive family education, parents may support consistent study-rest routines and self-discipline, which may correlate with less depletion of self-regulatory resources linked to academic stress and excessive Internet use.

### 4.6 Limitations and future directions

The study still has some limitations. First, this study adopted only online questionnaires as the sole data collection tool, leading to a monotonous empirical design without multi-angle in-depth data exploration. More importantly, the cross-sectional research design prevents robust causal inference across all study variables. While our hypothesized model positions Internet addiction as a variable correlated with higher academic stress, academic strain may also be statistically linked to compulsive Internet use as an avoidant coping tactic. Given that cross-sectional data cannot identify the temporal sequence of these two variables or measure the magnitude of their mutual feedback cycle, subsequent research should adopt longitudinal panel surveys or controlled experimental designs. Such rigorous methodologies can systematically unpack the bidirectional reciprocal linkage between Internet addiction and academic stress to validate and extend the current results. Second, this research only collects data from five universities within Anhui Province, and the obtained sample fails to represent nationwide populations. Such regional constraints impair the generalizability of research outcomes to a certain degree. Accordingly, subsequent studies are suggested to adopt nationally representative samples alongside a multicenter research framework.

## 5 Conclusion

This study examined the association between Internet addiction and academic stress among college students, with social anxiety and self-control tested as mediators. The findings showed that academic stress was positively associated with Internet addiction and social anxiety, and negatively associated with self-control. Internet addiction showed a direct statistical association with academic stress, alongside two parallel indirect pathways statistically mediated by social anxiety and self-control, and one sequential indirect pathway running through social anxiety to self-control. These findings are consistent with self-regulation theory, the limited-resource model of self-control, and Conservation of Resources Theory, which suggest that social anxiety and reduced self-regulatory resources may be important psychological correlates of academic stress. Crucially, all results are derived from cross-sectional correlational data. The observed mediating pathways should not be interpreted as evidence of causal relationships. We cannot draw conclusions regarding directional causal effects among variables.

These findings provide theoretical insight into the statistical association between Internet addiction and academic stress and suggest that social anxiety and self-control may be important factors to consider when examining this relationship. From a practical perspective, universities may deliver regular psychological counseling and social skills training; such resources may correlate with lower social anxiety, stronger self-regulation, and healthier online behavior. In addition, universities may consider balancing academic workload, strengthening academic support services, and expanding access to campus mental health resources to help students better cope with academic stress at the institutional level. Such multi-level campus support may correlate with fewer negative academic and psychological outcomes linked to Internet addiction.

Overall, this study indicates that Internet addiction, social anxiety, self-control, and academic stress are closely related among college students. The observed pattern suggests that greater attention should be given to both emotional and self-regulatory factors in prevention and intervention efforts. Future research should adopt longitudinal or experimental designs to clarify temporal ordering and test potential causal mechanisms linking these variables.

## Data Availability

The original contributions presented in this study are included in this article/Supplementary material, further inquiries can be directed to the corresponding author.

## Footnotes

1
www.wjx.cn
